# Botulinum toxin therapy of cervical dystonia: comparing onabotulinumtoxinA (Botox^®^) and incobotulinumtoxinA (Xeomin^®^)

**DOI:** 10.1007/s00702-013-1076-z

**Published:** 2013-08-04

**Authors:** Dirk Dressler, Pawel Tacik, Fereshte Adib Saberi

**Affiliations:** Movement Disorders Section, Department of Neurology, Hannover Medical School, Carl-Neuberg-Str. 1, 30625 Hannover, Germany

**Keywords:** Botulinum toxin, Therapeutic use, Cervical dystonia, Treatment duration, Interinjection intervals, IncobotulinumtoxinA (Xeomin^®^), OnabotulinumtoxinA (Botox^®^)

## Abstract

Several botulinum toxin (BT) drugs are licensed for the treatment of cervical dystonia (CD). We wanted to compare the efficacy and the potency labelling of incobotulinumtoxinA (Xeomin^®^) and onabotulinumtoxinA (Botox^®^) by analysing the duration of their therapeutic effect in a cross-over study. For this we studied 40 CD patients (26 females, 14 males, age at therapy onset 52.6 ± 12.0 years, duration of dystonia at therapy onset 10.0 ± 9.2 years, Tsui score 9.1 ± 3.9) who first received Botox^®^ and then Xeomin^®^ for at least 4 injection series each. BT doses were exchanged based on a 1:1 conversion ratio. Altogether 1,101 treatment cycles were evaluated. For each patient 27.5 ± 13.1 treatment cycles were recorded. Patients received 18.4 ± 12.4 treatment cycles with Botox^®^ and 9.2 ± 4.5 with Xeomin^®^. The treatment duration (TD) throughout the treatment course was 11.3 ± 1.0 weeks (Botox^®^ 11.2 ± 1.1 weeks, Xeomin^®^ 11.4 ± 1.3 weeks). The interinjection interval (II) throughout the treatment course was 14.8 ± 1.9 weeks (Botox^®^ 14.7 ± 1.6 weeks, Xeomin^®^ 15.0 ± 2.2 weeks). The mean difference between Botox^®^ and Xeomin^®^ was 0.3 weeks for TD (two-sided 95 % confidence interval [−0.3; 0.9]) and 0.5 weeks for II (two-sided 95 % confidence intervals [−0.4; 1.4]). The confidence intervals of both parameters were within the predefined therapeutic equivalence range set to ±1.5 weeks, thus indicating similar efficacy of both BT drugs. Having based the exchange of Botox^®^ and Xeomin^®^ on a conversion factor of 1:1 our data confirm previous findings of an identical potency labelling of both products, thus allowing comparisons of efficacy, adverse effects and costs.

## Introduction

Botulinum toxin (BT) is generally accepted as therapy of choice for the treatment of cervical dystonia (CD). Several BT drugs are licensed for this indication. OnabotulinumtoxinA (Botox^®^) was the first drug used for CD treatment. It is generally recognised as the gold standard for comparative studies. IncobotulinumtoxinA (Xeomin^®^) was introduced recently and features immunological advantages (Dressler 2012). Whenever a drug becomes available it is interesting to compare its efficacy with existent ones. For BT drugs, additionally, the issue of potency labelling needs to be raised (Marsden 1993). We, therefore, wanted to compare the efficacy as well as the potency labelling of Botox^®^ and Xeomin^®^ by analysing the duration of their therapeutic effect in a cross-over study.

## Methods

### Design

The study followed a prospective open label cross-over design and was approved by the local ethical committee. All patients in our BT outpatient clinics receiving BT therapy for CD were monitored for the treatment duration (TD) and interinjection intervals (II). Those who were initially treated with Botox^®^ and were later switched to Xeomin^®^ were randomly included in this study until *n* = 40 were reached.

### Definitions

Injection series is the set of BT injections given at one appointment. Treatment cycle is the treatment between two subsequent BT injection series. Treatment course is the time between the first and the last injection series recorded in one patient. TD describes the time between the BT application and the onset of the decrease of the therapeutic effect as reported by the patient. II describes the time between two subsequent BT injection series.

### Patients

All patients in our BT outpatient clinics fulfilling the following inclusion criteria were included in the study: (1) BT therapy with Botox^®^ for CD. CD severity was measured on the Tsui scale (Tsui and Calne 1988). Additional dystonia manifestations including periocular, oromandibular and shoulder girdle muscles were allowed. (2) Botox^®^ application for ≥4 injection series. (3) Exchange of Botox^®^ to Xeomin^®^. (4) Subsequent BT therapy with Xeomin^®^ for ≥4 injection series.

### BT therapy

BT therapy was performed with Botox^®^ (Pharm-Allergan, Ettlingen, Germany) and Xeomin^®^ (Merz Pharmaceuticals, Frankfurt/M, Germany) in dilutions of 100 MU per 2.5 ml of 0.9 % NaCl/H_2_O. Botox^®^ doses were exchanged to Xeomin^®^ doses using a 1:1 conversion ratio (Dressler et al. 2012). Mixtures of Botox^®^ and Xeomin^®^ were not applied. Target muscle selection was based upon analysis of the individual dystonic muscle involvement. Target muscles of the neck included sternocleidomastoideus, splenius capitis, semispinalis capitis, trapezius (cervical part), trapezius (horizontal part), scalenii and levator scapulae muscles. The BT dosing for each target muscle followed internationally accepted guidelines as previously published (Benecke et al. 2003). Regular II was 12–14 weeks. Re-injections were modified according to the patient’s needs or requests. Spontaneous postponement by the patient was allowed. Under exceptional circumstances II could be reduced to 8 weeks.

### Parameters

Demographic data collected for each patient included age at therapy onset, sex, duration of dystonia at therapy onset and Tsui score. Treatment data included II between all injection series, total BT dose given at each injection series and general remarks. Outcome parameter was the TD of each injection series as reported by the patient at each re-injection appointment.

### Statistics

Two-sided 95 % confidence intervals were calculated to assess the therapeutic equivalence for TD and II. The predefined equivalence range for both TD and II was set to ±1.5 weeks.

## Results

### Patients

Altogether 40 patients (26 females, 14 males, age at therapy onset 52.6 ± 12.0 years, duration of dystonia at therapy onset 10.0 ± 9.2 years) were evaluated in the study. CD severity was 9.1 ± 3.9 on the Tsui scale.

### General

Altogether 1,101 treatment cycles were evaluated in the 40 patients monitored in this study. For each patient 27.5 ± 13.1 treatment cycles were recorded. This is equivalent to an average treatment course of approximately 7 years. The maximal number of treatment cycles was 66 covering a treatment course of approximately 17 years, and the minimal 10 covering a treatment course of approximately 2.5 years. Patients received 18.4 ± 12.4 treatment cycles with Botox^®^ and 9.2 ± 4.5 with Xeomin^®^. The average BT dose applied was 295.7 ± 96.1 MU of either Botox^®^ or Xeomin^®^.

### Treatment duration

**Fig. 1:**
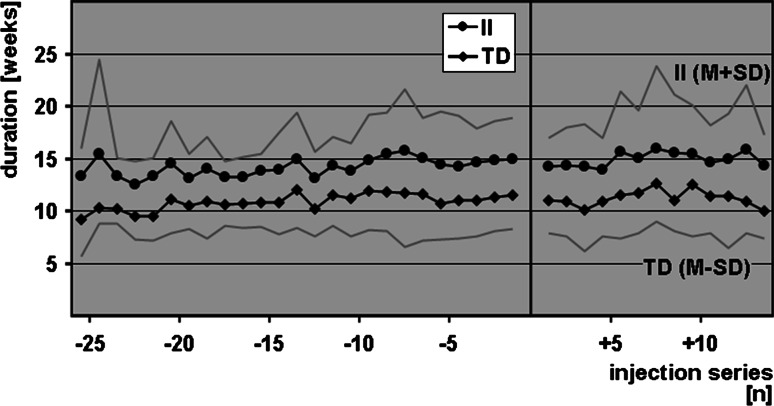
Interinjection intervals and treatment durations of all 40 patients studied. Only data points deriving from four or more patients are included. The *bar* indicates the time when Botox^®^ treatments were switched to Xeomin^®^ treatments using a 1:1 conversion ratio.* M+SD* mean plus standard deviation, *M-SD* mean minus standard deviation

TD throughout the treatment course was 11.3 ± 1.0 (minimum 7.8 ± 1.4, maximum 21.0 ± 3.9) weeks. When patients received Botox^®^ TD was 11.2 ± 1.1 (minimum 7.9 ± 1.5, maximum 20.5 ± 4.3) weeks, and under Xeomin^®^ it was 11.4 ± 1.3 (minimum 7.8 ± 1.4, maximum 22.3 ± 2.2) weeks. The mean difference between Botox^®^ and Xeomin^®^ was 0.3 weeks (two-sided 95 % confidence interval [−0.3; 0.9]). The confidence interval was within the therapeutic equivalence range of ±1.5 weeks, thus indicating similar efficacy of both BT drugs. Figure 1 shows the average of all TD before and after the patients were switched from Botox^®^ to Xeomin^®^. Only data points with four or more contributing patients are included. There is no obvious increase or decrease of TD throughout the treatment course. In 30 out of 40 patients (75 %), we found TD ≤12 weeks; in 12 out of the 40 patients (30 %), TD ≤10 weeks.

### Interinjection intervals

II throughout the treatment course was 14.8 ± 1.9 (minimum 11.3 ± 1.3, maximum 26.2 ± 7.7) weeks. When patients received Botox^®^ II was 14.7 ± 1.6 (minimum 11.5 ± 1.5, maximum 24.9 ± 5.5) weeks, and under Xeomin^®^ it was 15.0 ± 2.2 (minimum 10.4 ± 1.1, maximum 26.5 ± 1.3) weeks. The mean difference between Botox^®^ and Xeomin^®^ was 0.5 weeks (two-sided 95 % confidence intervals [−0.4; 1.4]). The confidence interval was within the therapeutic equivalence range of ±1.5 weeks, thus, again, indicating similar efficacy of both BT drugs. Figure 1 shows the average of all II before and after the patients were switched from Botox^®^ to Xeomin^®^. Only data points with four or more contributing patients are shown. There is no obvious increase or decrease of II throughout the treatment course.

## Discussion

With 1,101 injection series evaluated and 27.5 ± 13.1 treatment cycles (appr. 7 years) monitored in each patient, this study is one of the most comprehensive studies of its kind. Documentation of all patients by only two observers (DD, FAS) throughout the complete observation period is another unique feature of this study.

With II of 14.8 ± 1.9 weeks patients included in this study were treated according to current guidelines recommending II of more than 12 weeks to avoid BT antibody formation. Subsequently, none of the patients studied developed BT antibody-induced therapy failure (Dressler 2004) during the treatment course.

TD was 11.3 ± 1.0 weeks. Throughout the treatment course TD does not show increase or decrease confirming previously described lack of tachyphylaxia and induction in BT therapy. Long-term reproducibility of the BT response with constant TD is one of the most surprising features of BT therapy. In 30 out of 40 patients (75 %), we found TD ≤12 weeks; in 12 out of the 40 patients (30 %), TD ≤10 weeks. This confirms the need to consider decreasing II to avoid considerable time periods of suboptimal treatment.

Comparison of the therapeutic response to Botox^®^ and to Xeomin^®^ produced TD of 11.2 ± 1.1 weeks and 11.4 ± 1.3 weeks, respectively, and II of 14.7 ± 1.6 weeks and 15.0 ± 2.2 weeks, respectively. With two-sided 95 % confidence intervals of both parameters falling within the therapeutic equivalence range set to ±1.5 weeks, similar efficacy of both BT drugs is indicated. Having based the switch from Botox^®^ to Xeomin^®^ on a conversion factor of 1:1 confirms previous findings of an identical potency labelling of both products (Benecke et al. 2005; Dressler 2009; Dressler et al. 2012), thus allowing comparisons of efficacy, adverse effects and costs.
